# A basaloid carcinoma with multilocular thymic cyst mimicking a mediastinal teratoma

**DOI:** 10.1186/s13019-024-02712-z

**Published:** 2024-04-10

**Authors:** Chen Su, Xiaobo Zhu, Qiang Wang, Junjie Zhang

**Affiliations:** 1 Department of Cardiothoracic Surgery, Wujin Hospital Affiliated with Jiangsu University, No.2 North Yongning Road, Changzhou, 213000 China; 2 Department of Cardiothoracic Surgery, Wujin Clinical college of Xuzhou Medical University, No.2 North Yongning Road, Changzhou, Jiangsu Province China

**Keywords:** Thymic basaloid carcinoma, Multilocular thymic cyst, Mediastinal teratoma, Case report, Thymic tumor

## Abstract

This case report details a rare thymic basaloid carcinoma initially misinterpreted as a mediastinal teratoma, underscoring the diagnostic challenges posed by such tumors. A 71-year-old female presented with an asymptomatic anterior mediastinal tumor discovered incidentally during a routine health examination. Surgical intervention, followed by pathological and immunohistochemical analysis including CK-pan, p63, p40, and CD117 molecules, led to a definitive diagnosis of basaloid carcinoma of the thymus. This case highlights the critical importance of differential diagnosis in mediastinal lesions, especially those presenting with multilocular thymic cysts on chest CT. The subxiphoid video-assisted thoracoscopic surgery enabled complete tumor resection with minimal trauma and favorable postoperative outcomes. The patient opted against further radiotherapy or chemotherapy and she has survived for over eight months without recurrence. This case report contributes to the growing understanding of thymic basaloid carcinoma, a rare and potentially aggressive thymic carcinoma subtype. It emphasizes the necessity for precise surgical techniques and enhanced diagnostic acumen among cardiothoracic surgeons and oncologists.

## Introduction

Thymic tumors are particularly intricate due to their potential to manifest in a wide range of histological subtypes, each presenting distinct clinical and pathological characteristics [1]. One such rare and challenging variant is thymic basaloid carcinoma (TBC), which poses diagnostic dilemmas due to its resemblance to other mediastinal masses [2]. In this case report, we present a perplexing clinical scenario involving a patient initially suspected of having a mediastinal teratoma. The patient underwent surgical intervention followed by comprehensive pathological examination and immunohistochemical analysis. These investigations ultimately led to a definitive diagnosis of TBC. This case report aims to enhance the differential diagnostic capabilities of cardiothoracic surgeons and oncologists regarding mediastinal space-occupying lesions presenting with heterogeneous density or multilocular thymic cyst (MTC) on chest computed tomography (CT). Furthermore, despite a preoperative misdiagnosis, the subxiphoid approach for resection of this type of carcinoma yielded favorable outcomes.

## Case report

**Fig. 1:**
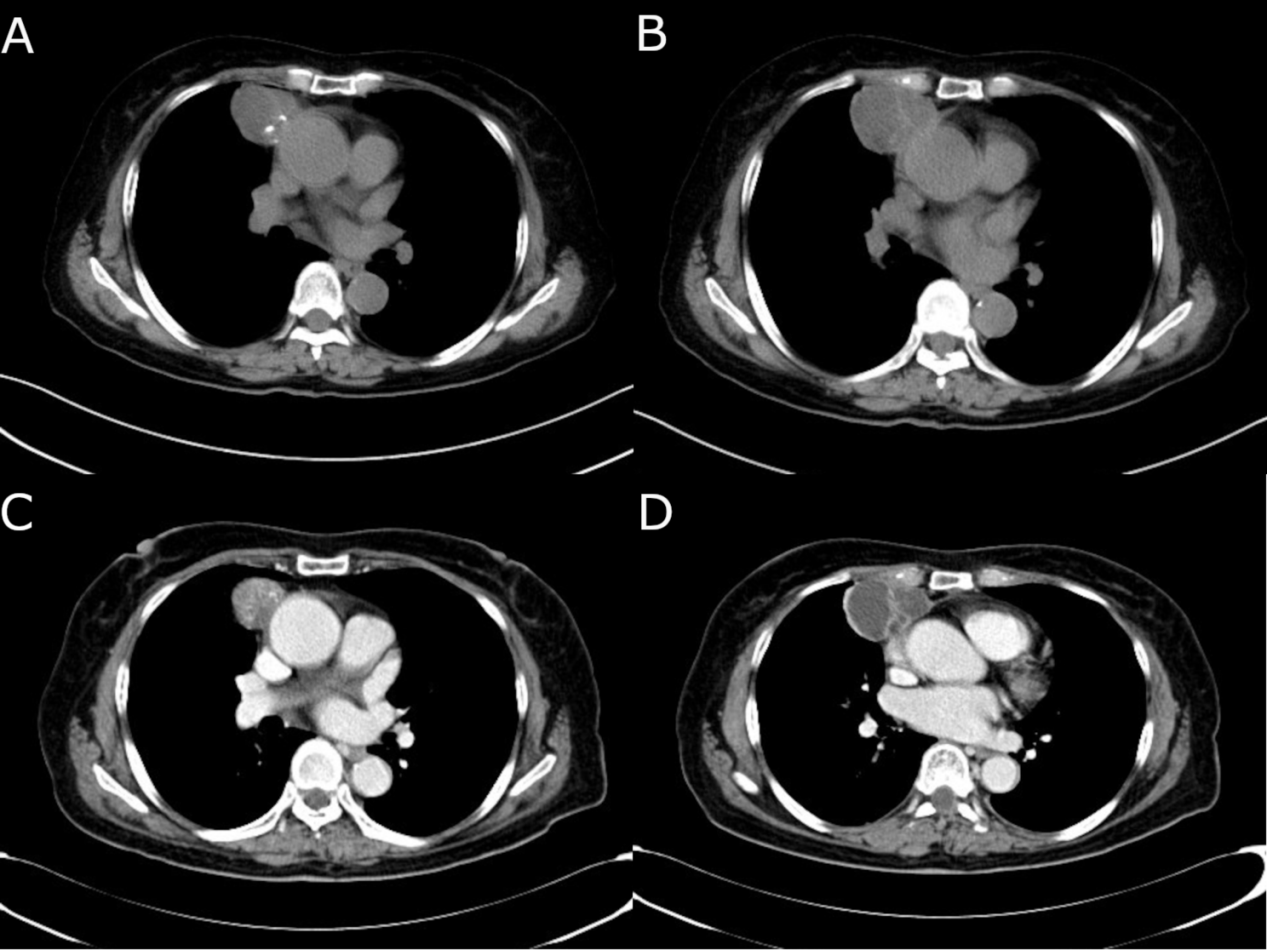
(**A** and **B**) A non-contrast chest CT scan revealed a mass in the right anterior mediastinum, measuring approximately 5.0 cm × 4.0 cm, with punctate calcifications noted within. The mass was closely adjacent to the ascending aorta and the right lung lobes. (**C** and **D**) Contrast-enhanced chest CT indicated that the mass was cystic and solid, displaying multiple separated cystic spaces, containing soft tissue, calcifications, and cystic components. The mass was well-demarcated from the surrounding tissues and organs

**Fig. 2:**
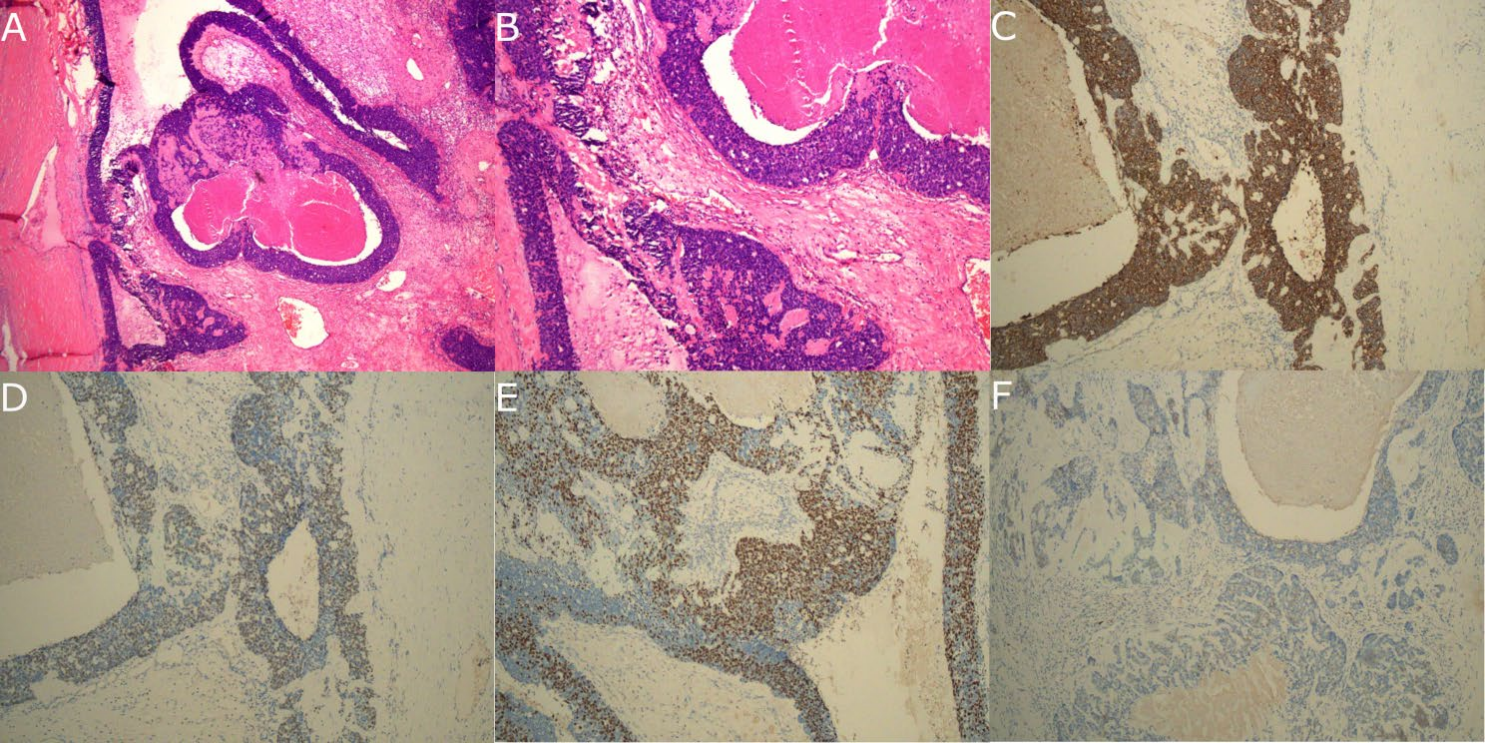
(**A**) H&E staining revealed that the inner layer of the tumor cyst wall was composed of basaloid tumor cells arranged in a palisade pattern, with focal comedo necrosis observed. The tumor tissue exhibited a mixed distribution of cystic and nested patterns. The tumor cells were moderately small with a high nucleus-to-cytoplasm ratio, displaying uniform basophilic staining (×40). (**B**) H&E staining indicated an orderly arrangement of tumor cells. No significant tumor cells were observed within the cyst cavity, and there were no notable nodules in the cyst wall. Outside the cyst cavity, there were no signs of tumor cells exhibiting papillary projections or invasive characteristics (×100). (**C–F**) **(**Immunohistochemistry), (**C**) showed strong positive reactivity of CK-pan. (**D**) showed strong positive reactivity of p63. (**E**) showed strong positive reactivity of p40. (**F**) showed weak positive reactivity of CD117

A 71-year-old female patient was incidentally discovered to have an unidentified tumor in the anterior mediastinum during a routine health examination one month prior. This incidental finding led to the patient being admitted to our hospital for further evaluation and management. Upon admission, she remained asymptomatic with no complaints of discomfort. Her medical history was notable only for hypertension and a previous surgery for an intraspinal meningioma. Vital signs on arrival were unremarkable except for a mild elevation of blood pressure. Tumor markers including alpha-fetoprotein (AFP), human chorionic gonadotropin (hCG), and carcinoembryonic antigen (CEA), as well as inflammatory markers such as C-reactive protein (CRP), procalcitonin (PCT), and interleukin-6 (IL-6), were all within normal ranges. A non-contrast chest CT scan revealed a tumor located in the right anterior mediastinum, measuring approximately 5.0 cm × 4.0 cm. The tumor exhibited heterogeneous internal density with punctate calcifications (Fig. 1A and B). Subsequent contrast-enhanced CT scans showed the tumor to be cystic and solid in nature, containing multilocular cysts. The tumor is characterized by well-defined borders, and is closely adjacent to the right lung lobes and pericardial tissues, with no apparent signs of invasion into the aorta (Fig. 1C and D). Additionally, there was no evident enlargement of the mediastinal lymph nodes. To further diagnose, we recommended a PET-CT examination for the patient. However, as an elderly individual living alone with financial difficulties, the patient was unable to afford the costly procedure. Based on the findings of the contrast-enhanced CT, which showed soft tissue, cystic, and calcific components within the tumor, we preliminarily concluded that the likelihood of a mediastinal teratoma was high. Most mediastinal teratomas are benign, and given the tumor’s location in the anterior mediastinum and its well-defined borders, we believed that a subxiphoid approach would allow complete resection of the tumor, thereby reducing potential complications for the patient. During the surgical procedure, the tumor was observed to have a soft texture with an intact capsule, containing opaque fluid and some necrotic tissues. Apart from a dense adhesion to the right middle lung lobe, the other margins of the tumor were well-defined from adjacent tissues and easily separable. We successfully performed *en bloc* tumor resection and thymectomy via thoracoscopic surgery. Due to difficulties in separating the tumor from the right middle lung, a stapler was utilized to execute a combined partial lobectomy. Hematoxylin and Eosin (H&E) staining indicated basaloid carcinoma (Fig. 2A and B). Immunohistochemical analysis revealed strong positive reactivity for CK-pan, p63, and p40, and a weak positive reactivity for CD117 (Fig. 2C and F). The tumor was adherent to but did not invade the lung. The final pathological staging was determined as Masaoka Stage II. Postoperatively, the patient recovered smoothly. We recommended further radiotherapy, but she strongly refused and opted to self-administer some traditional Chinese medicines. Six months later, a CT scan indicated no local recurrence of the tumor, and the patient has now survived for eight months.

## Discussion

The thymus is a critical organ for T-cell maturation and is capable of producing a spectrum of neoplasms, including thymic carcinoma with various histological subtypes. Thymic carcinoma is a rare epithelial-derived malignancy, accounting for approximately 20% of all thymic epithelial tumors [3]. Among these, TBC represents a unique and exceedingly rare subtype. Although traditionally classified as a low-grade neoplasm, TBC has demonstrated potential for aggressive clinical behavior and notable mortality risk [2]. Its rarity and overlapping clinical and radiological features with other mediastinal tumors, such as teratomas and lymphoma, further complicate its accurate diagnosis and timely management.

To our knowledge, no more than 30 cases of TBC were reported in the English literature [4, 5]. A case series published in 2009 reported the clinical pathology and immunohistochemical characteristics of 12 cases of TBC [5]. Building on this, we have compiled and summarized most of the previously published cases, as detailed in Table 1. Incorporating previously published cases and case series, most patients with TBC were incidentally diagnosed without any specific complaints. A minority of patients may present symptoms such as chest pain and dyspnea due to tumor compression [6, 7]. The average age of onset was around 60 years, with a male to female ratio of approximately 2:1. In nearly all patients, tumor markers were within normal limits. Notably, one case exhibited a significant elevation in CEA levels, which may be attributed to concurrent hepatic metastases, thereby indicating the non-specific nature of tumor markers in this context. Although TBC is classified as a low-grade malignancy, some patients present at diagnosis with vascular, neural, or surrounding tissue invasion, as well as metastases to the liver and lung [8–10]. Consequently, this disease still exhibits a degree of aggressiveness, underscoring the need for clinicians to enhance their diagnostic capabilities for this condition.

In this case, we observed MTC changes on the enhanced chest CT, characterized by punctate calcifications and heterogeneous densities. The patient’s refusal to undergo PET-CT posed a diagnostic challenge. The phenomenon of cystic changes within anterior mediastinal tumors have been well-documented, encompassing a diverse array of neoplasms intimately associated with MTC changes. This encompasses an array of primary mediastinal tumors, including thymomas, germ cell tumors, and lymphomas [11, 12]. For example, mediastinal teratomas, which are commonly located in the anterior mediastinum, typically exhibit cystic and solid components, including soft tissue, calcifications, and fat, thereby showing heterogeneous density [13]. Furthermore, instances where MTC changes coexist with metastatic malignancies have also been well-established [14]. In the absence of PET-CT, fine-needle aspiration biopsy (FNAB) is an alternative diagnostic approach. However, previous cases indicate limitations of FNAB, including incomplete tumor tissue retrieval and false negatives [15, 16]. In cases diagnosed with FNAB, the tumors did not exhibit MTC changes. It is hypothesized that in tumors with MTC changes, where the tumor cells are located in the cystic lining and the interior consists of fluid or necrotic tissue, FNAB might not yield a high tumor detection rate. Our aggressive diagnostic approach led to a preoperative misdiagnosis in this case.

In elucidating the pathogenesis of MTC changes in tumor and tumor-like settings, two prevalent theories have emerged. One theory suggests a transformation of the lining epithelium of pre-existing MTC, accounting for the observed continuity between cyst lining and tumor. The other hypothesis, contrasting the first, postulates cystic changes as a hyperplastic reaction of thymic epithelium to specific tumor antigens, culminating in the dilation of Hassall corpuscles [17]. Brown JG et al. summarized 12 cases, founding MTC changes in only one patient, which constitutes approximately 8.3% — a proportion comparable to that in our summary in Table 1. Their description of over ten earlier published cases showed about 50% MTC changes [5]. These data indicate a weak association of MTC changes with thymic basaloid carcinoma, contributing to the diagnostic challenges of the disease.

Before surgery, it would have been preferable to evaluate the tumor’s standardized uptake value using PET-CT, but studies suggest that PET-CT is not routinely recommended for assessing thymic tumors, as other types of mediastinal tumors and thymic hyperplasia can also exhibit high metabolic activity [18]. MRI can assess the infiltration of mediastinal tumors into surrounding fat, which is beneficial for planning appropriate surgical interventions, although this was not performed in our case. Analysis of previous cases indicated that surgical treatment remains the preferred method for TBC Even in cases at Masaoka stage IVb, neoadjuvant chemoradiation can be employed to downstage the tumor, allowing for subsequent complete resection [19]. The median sternotomy has been the most commonly used incision in previous cases, providing excellent exposure to anterior mediastinal tumors and facilitating extensive tumor resection. However, with advancements in minimally invasive surgical techniques, resection of anterior mediastinal tumors via a median sternotomy is no longer the only approach. In this case, with the tumor located on the right side of the mediastinum, a right lateral intercostal video-assisted thoracoscopic surgery (VATS) was considered. However, an alternative viable surgical pathway, the subxiphoid VATS, was chosen. Studies have demonstrated that compared to the lateral intercostal thoracic approach, the subxiphoid approach offers advantages in reducing intraoperative bleeding, postoperative hospital stay, postoperative thoracic drainage, and in lowering postoperative pain scores and analgesic medication usage [20]. The subxiphoid approach provides enhanced bilateral thoracic visualization, more thorough thymectomy, reduced trauma, and superior cosmetic outcomes, proving to be a safe and feasible minimally invasive surgical technique [21]. An additional rationale for opting for the subxiphoid approach in this case was the well-defined margins of the tumor and its lack of invasion into major blood vessels and other critical organ tissues. The subxiphoid VATS allows for the complete resection of the tumor while minimizing patient trauma and reducing the risk of complications. Certainly, the subxiphoid approach does have certain drawbacks. It is not advisable in cases where the tumor is large, or when it is intricately associated with complex anatomical structures such as the heart, major blood vessels, superior vena cava, or the brachiocephalic vein. Although there has been case report of successful complete resection of the large tumor via the subxiphoid approach, this pathway can increase the risk of intraoperative bleeding and the necessity for conversion to open thoracotomy [22].

Pathology and immunohistochemistry are the gold standards for diagnosing TBC. In this case, the tumor cells exhibited a classic nesting pattern of growth. The basaloid cells lining the cysts were arranged in a palisade pattern, forming nests of varying sizes. Focal comedo necrosis was observed internally, with no nodules or papillary projections noted within the cyst walls, and there were no evident signs of tumor invasion beyond the cystic structure. In certain pathological observations, basaloid carcinoma exhibits tendencies for squamous, glandular, and sarcomatoid differentiation. At the periphery of the tumor nests, small glandular structures and lumina are visible, with tumor cells exhibiting finger-like projections invading the stroma. Additionally, ruptured basaloid glands with mucin spilling into the interstitium are also observed [5]. In immunohistochemistry, the positivity of CK-pan, p63, and p40 is consistent with the tumor originating from thymic epithelial cells. Regarding postoperative radiochemotherapy for TBC, there is currently no consensus, and treatment recommendations are derived from those for thymic carcinoma. For lesions with R0 resection, adjuvant radiotherapy can be considered, generally with a dosage of 40–50 Gy [23]. For lesions with R1 resection, it is necessary to increase the radiotherapy dosage and expand the treatment field appropriately [24]. The possibility of complete surgical resection of thymic carcinoma is a key factor affecting postoperative recurrence and survival of patients [24]. Chemotherapy alone should be recommended only for metastatic thymic carcinoma that is inoperable or ineligible for radiotherapy [25]. From the limited cases studied previously, it has been observed that although TBC possesses a certain degree of invasiveness and metastatic potential, its overall prognosis remains favorable. The longest postoperative survival reported in a patient reached 11 years [6]. In our case, despite the intraoperative finding of tumor adhesion to the lung, pathology did not indicate tumor invasion, and the staging remained at Masaoka II. The patient did not receive postoperative radiotherapy or chemotherapy, and the specific prognosis requires a longer follow-up period for determination.

## Conclusion

This case of TBC highlights the importance of differential diagnosis in mediastinal lesions with MTC changes. The unique aspects of this case lie not only in its contribution to the growing accumulation of similar cases but also in highlighting the necessity for heightened diagnostic acumen among clinicians. In cases where complete resection of thymic malignancies is achievable, the subxiphoid VATS represents a commendable minimally invasive option.

**Table 1:** Characteristics of TBC in most previous English case reports (excluding the case series of Brown JG et al.)

Study	Age and sex	Complain	Tumor makers	Maximum diameter (mm)	CT (margin, attenuation, calcification, MTC)	FNAB	Invasion or metastasis	Management	Staging	Immunohistochemistry (positive)	Prognosis
Fukunaga A [26]	68-M	Discovered incidentally	Normal	38	Well-defined, heterogeneous, no, no	No	No	TR + TM, MS	II	CD5, Bcl-2, CD117, p63	Survived>24 m, no recurrence
Miura S [27]	53-M	Discovered incidentally	NA	70	Ill-defined, heterogeneous, yes, no	Yes	Sternum and pleural	Chemotherapy	IVa	CK5, CD117, CD5, Bcl-2	Survived>10 m, no progression
Kawashima O [15]	58-M	Discovered incidentally	Normal	60	NA, heterogeneous, no, yes	Yes (insufficient)	No	TR + radiotherapy, MS	II	CK-pan, Ber-EP4	Survived>25 m, no recurrence
Posligua L [6]	65-M	Discovered incidentally	NA	80	Well-defined, heterogeneous, no, no	Yes	No	TR + TM + LND + radiotherapy, MS	II	CK-pan, CD5, CD44, NSE	Survived>16 m, no recurrence
Posligua L [6]	50-M	Shortness of breath	NA	135	NA	Yes	No	TR + TM, MS	II	CK-pan, CD5, NSE	Survived>11 y, no recurrence
Posligua L [6]	73-M	NA	NA	NA	NA	No	Pericardium	TR + TM + radiotherapy, MS	III	CK-pan, CD5, NSE	Survived>33 m, no recurrence
Phen S [28]	57-M	Neck pain	CEA	85	Ill-defined, heterogeneous, yes, no	Yes	Liver	Chemotherapy	IVb	CD5, CD117, PAX8, p63	Died
Tagawa T [19]	61-F	Discovered incidentally	NA	NA	Ill-defined, heterogeneous, no, no	Yes	Lung	Neoadjuvant chemoradiotherapy + TR + TM	IVb	CD5	Survived>11 m, no recurrence
Lee ACH [7]	63-M	Chest pain	NA	71	Well-defined, heterogeneous, no, no	Yes	Lung	TR + CR + radiotherapy, MS	III	CK-pan, p63, p40	Survived>10 m, no recurrence
Suemitsu R [4]	72-M	Discovered incidentally	Normal	NA	Ill-defined, heterogeneous, no, yes	No	Lung, vessels and pericardium	TR + CR + radiotherapy, MS	III	CD5	NA
Matsuo T [16]	41-F	Chest pain	Normal	50	Well-defined, heterogeneous, no, no	Yes (insufficient)	Liver	TR + CR, MS	IVb	NA	Survived>24 m, recurrence
Sakakura N [29]	72-M	Discovered incidentally	NA	65	Well-defined, heterogeneous, no, no	No	No	TR + TM + CR, MS	II	CD5, CD117, Bcl-2	Survived>36 m, recurrence
Manthri S [2]	61-F	Discovered incidentally	NA	65	NA	Yes	Liver	Chemotherapy	IVb	CK-pan, CD5, CD117, GATA3	NA
Siddiqui S	58-F	Chest pain	NA	72	Well-defined, heterogeneous, no, no	Yes	Lung, pericardium	TR + CR + LND +chemoradiotherapy, MS	IVb	NA	Survived>6 m, no recurrence
Buero A	46-F	Chest pain	Normal	50	Ill-defined, heterogeneous, no, no	No	Sternum, trachea and vessels	Chemoradiotherapy	III	CK-pan, p63	Survived>24 m, no progression

M, male; F, female; NA, not available; TR, tumor resection; TM, thymectomy; MS, median sternotomy; LND, lymph node dissection; CR, combined resection (including lung, pericardium); m, months; y, years

## Data Availability

No datasets were generated or analysed during the current study.
